# Progress in Neurology

**Published:** 1903-12-12

**Authors:** 


### Progress in Neurology.

**Epilepsy.**—In a discussion of the subject of the epileptic aura, Spratling[1] said that it was present in about 45 per cent. of cases. It is generally believed that it originates in the central nervous system and is referred to the periphery. The various forms it takes can be grouped under four heads—viz., psychical, sensory, motor, and irregular auras. A psychical aura, which is present in about 4 per cent., might make its appearance in the form of a temporary disturbance of mind, for instance, a sudden acceleration of the imagination, or fear of a primary and unreasoning character. Sensory auras are chiefly visual, auditory, or epigastric, the latter consisting of a choking or deep-seated burning sensation in the epigastrium. Running or walking hurriedly before the onset of the fit is one of the most common forms of the motor aura. The more sudden and complete an attack the less likely is it to be preceded by an aura. Clark said that his view was that epilepsy is always a sensory disturbance of the cerebral cortex, with an explosive motor manifestation. A discharge of the sensory cells in the cortex not only constituted the aura or sensory disturbance, but as a secondary result the motor pyramidal cells were temporarily withdrawn from the inhibitory influence of these sensory cells, and were thus permitted to discharge freely and violently. There would be some form of aura in every case if the rate of cerebral diffusion of the discharge were slower or less complete than in the most severe cases. The explanation of the great frequency of visual auditory and epigastric auras lay in the extensive cortical representation of these functions. The longer and more elaborate the aura the less disposed is the motor cortex to react convulsively. Many recovering epileptics have only the aura of their former fits. If such patients carried a bottle of amyl nitrate or ammonium salts, and sniffed at it directly an aura was perceived, a fit could often be prevented.

General Paralysis of the Insane.—The researches of Bruce and of Robertson during recent years have begun to cast some doubt on the generally accepted view that general paralysis is due to syphilis. Bruce[2] had come to the following conclusions:—General paralysis is a disease directly due to poisoning by the toxines of bacteria, whose point of attack is through the gastric and intestinal mucous membrane. The poisoning is probably a mixed one, but the bacillus coli communis is apparently one of the noxious organisms. The result of treatment by means of serum taken from general paralysis in a condition of remission and injected subcutaneously points to the fact that some form of serum treatment is the proper one for this as yet incurable disease. Bruce's further observations led him to believe the bacillus coli infection to be a secondary one. Two patients were treated with antibacillus coli serum, but neither benefited, though a considerable leucocytosis developed. Three cases were then treated with small doses of a streptococcus pyogenes vaccine; in one there was no reaction in pulse temperature or leucocytosis, and there was no mental benefit, whilst in the two others there was a febrile reaction, hyperleucocytosis with a high polymorphonuclear percentage, and a distinctly beneficial action on the mental disorder. All these effects, however, passed off in about a week. Two patients were then injected with two c.c. of terebene, one did not react, the other had a sharp febrile reaction, hyperleucocytosis with a high percentage of polymorphonuclear cells, and considerable mental benefit, so that in two months he was discharged. It would thus appear that a sharp febrile attack plus a high polymorphonuclear leucocytosis is sufficient to arrest, at least temporarily, the course of general paralysis. Bruce holds that these experiments afford support to the view that the disease is due to the direct action of bacterial toxines, which when temporarily overpowered, allow nature to put into force the constant attempts at recovery which are so marked a feature of the disease. Robertson[3] is of opinion that the doctrine "no syphilis, no general paralysis" is marred by the fact that only a very small proportion of syphilitics become general paralytics or tabetics; and, secondly, by the fact that other conditions, such as chronic alcoholism, lead poisoning, and excessive meat diet favour the development of the disease; and, thirdly, by the fact that some cases, at least, have not had syphilis. He holds that the disease is the result of a chronic toxæmia dependent on the excessive development of bacteria not only in the alimentary canal but in the nasal tract and throat, and especially on the abundant growth of a diphtheroid bacillus. The recognised causes of general paralysis damage the leucoblastic tissue so that the defences of the body against the invasion of bacteria were diminished. The bacteria of the alimentary canal, throat, and nose, normally present as saprophytes, consequently assume a pathogenic character. The special infective agent is one resembling the Klebs-Löffler bacillus in culture and morphology, though not identical with it. It was found in 17 out of 20 cases of general paralysis in the nose and alimentary canal in post-mortem examinations, and in nine out of 10 cases from secretions from the nose and throat in living patients. Further, the organism, when introduced into the alimentary canal of rats, caused death, and changes closely resembling those of general paralysis were found in their brains.

Gastric Crises.—In a paper on this affection, Ewald,[4] of Berlin, gives some instances of how easily mistakes in diagnosis are made. Thus in one patient, a woman, the symptoms were at first attributed to pregnancy and were treated by cauterisation and dilatation of the cervix. The attacks continuing, a gastric ulcer with pylorospasm was diagnosed, and a gastro-enterostomy was done. As this had no effect, the crises were then attributed to the annexa of the uterus, and the patient was accordingly castrated, with no result. Subsequently she developed phthisis and died, when the autopsy revealed a sclerosis of the posterior columns of the spinal cord. The diagnosis is often difficult, as the signs of tabes may be but slight, *e.g.*, Argyll-Robertson pupils and loss of knee-jerks. Similar attacks of vomiting may occur in general spastic paralysis, disseminated sclerosis, and in compression of the spinal cord from a fractured vertebra. The paroxysmal character of the vomiting, and the normal performance of digestion during the intervals, show that no lasting functional or anatomical disturbance of the stomach takes place. In the intervals the acidity of the stomach contents is as a rule normal, during the attacks it is usually subnormal; so that the attack cannot be due, as was supposed, to hyperacidity. The only effectual treatment is the hypodermic injection of morphia. This is of course liable to set up a habit, but the risk must be faced.

Tabes Dorsalis.—According to Nageotte[5] tabetic degeneration consists essentially in a progressive destruction of the posterior roots; at an early stage the lesion is seen in the intra-medullary fibres of the roots only; when the changes are more advanced the extra-medullary portions are affected as well, though to a less degree. The diseased process thus spreads towards the trophic ganglion *i.e.*, the posterior root ganglion. Within the cord the short and medium fibres appear to be affected before the long fibres. The lesion is a slow atrophy which affects by preference certain kinds of fibres in each root. Meningeal changes exist, the pia mater is thick, and infiltrated with cells, chiefly lymphocytes. The vessels are always altered, especially the veins, their walls are infiltrated with lymphocytes, sometimes clustered together in nodules. The cerebro-spinal fluid shows the presence of a lymphocytosis. There is thus abundant evidence of a meningitis. Now this lymphocytosis may be found when the only symptom of the disease is an Argyll-Robertson pupil; Nageotte therefore thinks that the meningitis is not the consequence of parenchymatous changes in tabes, but that it precedes their development.

[1] Med. Rec., June 13, 1903. [2] Scottish Med. and Surg. Jour., June, 1903. [3] Lancet, Aug. 15, 1903. [4] Med. Rec., June 20, 1903. [5] Presse Médicale; Med. Chron., April, 1903.

(*To be continued.*)

